# Computational Investigation of the Thermoelectric Performance of Environmentally Friendly and Earth-Abundant SrZn_2_S_2_O

**DOI:** 10.1021/acsaem.5c03742

**Published:** 2026-01-24

**Authors:** Shipeng Bi, Katarina Brlec, Alexander G. Squires, David O. Scanlon

**Affiliations:** † Department of Chemistry, 4919 University College London, 20 Gordon Street, London WC1H 0AJ, United Kingdom; ‡ School of Chemistry, 1724 University of Birmingham, Edgbaston, Birmingham B15 2TT, United Kingdom

**Keywords:** thermoelectric materials, environmental friendliness, density functional theory, power factor, lattice thermal conductivity

## Abstract

Thermoelectric (TE) materials enable direct conversion between heat and electricity, allowing efficient recovery of waste heat, which accounts for nearly 50% of global energy consumption. Therefore, TE materials hold great potential for applications in waste heat recovery and sustainable energy technologies. Owing to the composition of earth-abundant and low-toxicity elements, as well as the presence of relatively heavy elements and mixed-anion characteristics, SrZn_2_S_2_O is considered a promising, environmentally friendly TE material. In this study, the TE performance of SrZn_2_S_2_O was investigated based on density functional theory (DFT) and compared with that of the prototypical mixed-anion oxide BiCuSeO. The calculated results show that SrZn_2_S_2_O exhibits a higher optimal average *p*-type power factor than that of BiCuSeO at 900 K, reaching 1150 μW m^–1^ K^–2^ compared with 770 μW m^–1^ K^–2^ for BiCuSeO. In addition, nanostructuring strategies can reduce the lattice thermal conductivity of SrZn_2_S_2_O by 40% or more in all crystallographic directions. This leads to a maximum *n*-type *ZT* value of 0.65 along the *b* direction and a maximum *p*-type *ZT* value of 0.77 along the *c* direction for SrZn_2_S_2_O.

## Graphical abstract

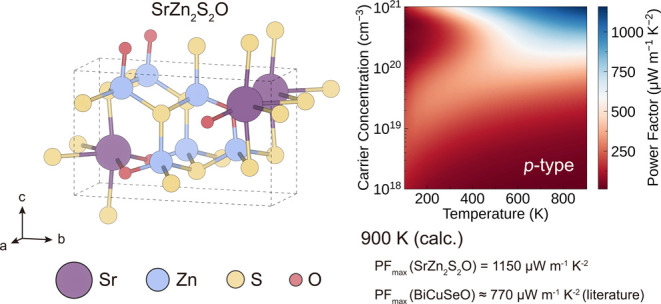

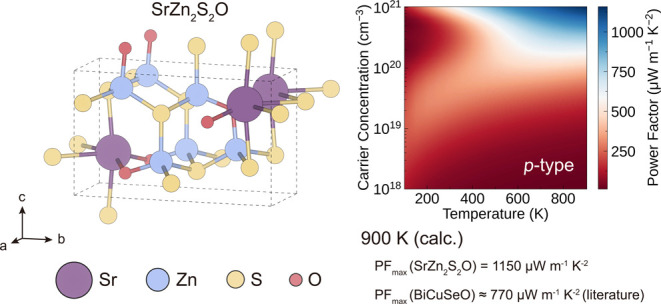

## Introduction

It is estimated that approximately 50% of the world’s total energy is dissipated as waste heat; thus, its recovery would greatly improve overall energy efficiency.^1^ Thermoelectric (TE) generators can directly convert waste heat into electrical energy by utilizing a temperature gradient. Therefore, TE energy conversion is regarded as a feasible and efficient approach to energy recovery. In this process, the temperature difference drives charge carriers to diffuse from the hot side to the cold side, generating an electrical voltage and enabling energy conversion. The performance of TE materials is commonly evaluated by the dimensionless figure of merit (*ZT*), which is defined as

1 $$\mathrm{ZT}=\frac{S^{2}\sigma T}{\kappa_{e}+\kappa_{l}}$$

where *S* is the Seebeck coefficient, σ is the electrical conductivity, *T* is the absolute temperature, and κ_e_ and κ_l_ represent the electronic and lattice thermal conductivities, respectively. In theory, an excellent TE material should possess a high Seebeck coefficient, high electrical conductivity, and low thermal conductivity. However, optimizing the *ZT* value is extremely challenging because these parameters are strongly interdependent. Increasing the charge carrier concentration (*n*) or reducing the charge carrier effective mass can enhance electrical conductivity but usually leads to a decrease in the Seebeck coefficient. Moreover, good electrical conductivity usually implies high electronic thermal conductivity.^2^ Therefore, it is essential to achieve an appropriate balance among these competing factors. Ideal TE materials can be understood through the *phonon-glass electron-crystal* (PGEC) concept,^3^ in which a material exhibits glass-like phonon transport while maintaining crystal-like electronic transport. Guided by this concept, a series of high-performance TE materials, such as Sn- and Pb-based chalcogenides, have been developed. Nevertheless, these materials often suffer from poor high-temperature stability, scarcity or toxicity of constituent elements, or limited conversion efficiency, which hinders their large-scale application.^4–6^

For the development of next-generation TE materials, not only is high conversion efficiency important, but also the abundance and environmental friendliness of the constituent elements must be taken into account. In this respect, oxide compounds are regarded as highly promising candidates for TE applications due to their excellent thermal stability in air, generally low toxicity, and abundance of resources.^7–9^ However, it should be noted that this perceived “abundance” mainly originates from the high natural abundance of oxygen, while some other constituent elements in oxides are not necessarily low-cost or readily available. Among *p*-type oxide TE materials, Co-based oxides have attracted extensive attention since Terasaki et al.^10^ reported that NaCo_2_O_4_ exhibits an outstanding power factor. For instance, single-crystalline Na_*x*_CoO_2‑δ_ shows a *ZT* value of 1.2 at 800 K, while another Co-based oxide, Ca_3_Co_4_O_9+δ_, achieves a *ZT* value of 0.9 at 1073 K after Tb and Bi doping.^11,12^ Although Co is not an abundant element, these studies have advanced the development of oxide TE systems. Meanwhile, increasing attention has been devoted to alternative oxide systems composed of more earth-abundant elements. For instance, Kamiya et al.^5^ investigated the *p*-type TE performance of the inverse-perovskite Ba_3_SiO and Ba_3_GeO, reporting *ZT* values of 0.84 (623 K, *n* ≈ 5.0 × 10^18^ cm^–3^) and 0.65 (523 K, *n* ≈ 2.7 × 10^19^ cm^–3^), respectively. However, theoretical calculations predict that further optimization of the carrier concentration at 600 K could yield maximum *ZT* values of 2.14 and 1.21 for Ba_3_SiO (*n* = 8.1 × 10^19^ cm^–3^) and Ba_3_GeO (*n* = 1.6 × 10^20^ cm^–3^), respectively. Therefore, precise control of the carrier concentration is crucial for further enhancing the TE performance of these materials.

For *n*-type oxide TE materials, ZnO has long been considered a promising candidate, because it is a common and inexpensive semiconductor. However, the intrinsic *ZT* value of ZnO is quite low until Ohtaki et al.^13^ significantly improved its performance through codoping with Al and Ga, which increased the *ZT* to 0.65 at 1247 K and brought ZnO back into the spotlight. Among the various *n*-type oxides investigated, SrTiO_3_ is one of the most representative systems. Donor-doped SrTiO_3_ exhibits a relatively high power factor,^14–16^ and its high melting point (2080 °C)^17^ endows it with excellent thermal stability at high temperatures. However, its relatively high lattice thermal conductivity severely limits its overall TE performance.^14,15,18^ The best TE performance of SrTiO_3_ reported so far was achieved using a codoping strategy with 10 mol % La and 10 mol % Nb, which exhibited a *ZT* value between 0.6 and 0.7 at 1000–1100 K.^19^ In addition, researchers have also attempted to explore its derivative phases; however, no satisfactory TE performance has yet been obtained.^20^ Another promising *n*-type oxide is CaMnO_3‑δ_ ceramics. Benefiting from the synergistic effects of Bi doping, which increases the electron concentration, and the CuO secondary phase at grain boundaries, which enhances carrier mobility, the material achieves a *ZT* value of 0.67 at 773 K.^21^ This *ZT* value remains the highest reported to date among perovskite oxide ceramics.

At present, the *ZT* values of oxide TE materials remain to be further improved, with their relatively low *ZT* values primarily being attributed to high thermal conductivity. Recently, SrZn_2_S_2_O has been proposed as a novel photocatalyst for water splitting.^22^ This compound crystallizes in the *Pmn*2_1_ space group and contains the heavy element Sr, which is expected to contribute to a low lattice thermal conductivity. In addition, SrZn_2_S_2_O incorporates two types of anions (S^2–^ and O^2–^), making it a mixed-anion compound. In recent years, the mixed-anion strategy has been recognized as an effective approach to achieving low lattice thermal conductivity, since the introduction of additional anions can enhance phonon scattering.^23,24^ Meanwhile, the constituent elements of SrZn_2_S_2_O are earth-abundant and environmentally friendly, giving the material potential advantages in the design of sustainable TEs. In this work, we employed density functional theory (DFT) calculations to investigate the electronic and phonon transport properties of SrZn_2_S_2_O and evaluate its TE performance. Furthermore, nanostructuring is a commonly employed strategy for enhancing TE performance. When the grain size is reduced to 10 nm, *n*-type SrZn_2_S_2_O exhibits a maximum *ZT* value of 0.65 along the *b* direction at 900 K, primarily due to its lower thermal conductivity. In contrast, the *p*-type SrZn_2_S_2_O achieves the highest *ZT* value of 0.77 along the *c* direction, which can be attributed to its superior power factor in that direction.

## Computational Details

All DFT calculations in this study were performed using the Vienna *Ab initio* Simulation Package (VASP).^25^ The interactions between the core and valence electrons were treated using the projector augmented-wave (PAW) pseudopotential method.^26,27^ The plane-wave cutoff energy was set to 500 eV. Since the conventional and primitive unit cells of SrZn_2_S_2_O are identical, a Γ-centered *k*-point mesh of 7 × 3 × 4 was employed to sample the Brillouin zone for the 12-atom unit cell. These computational parameters ensure that the total energy converges within 1 meV per atom. The convergence tests of the plane-wave energy cutoff and *k*-point mesh are provided in Section 1 of the Supporting Information.

During structural optimization, the Heyd-Scuseria-Ernzerhof hybrid functional (HSE06)^28,29^ and the Perdew–Burke–Ernzerhof functional revised for solids (PBEsol)^30^ within the generalized gradient approximation (GGA) were employed. Based on the HSE06-optimized structure, the electronic band structure, density of states (DoS), deformation potentials, wave function coefficients, and high-frequency dielectric constants were computed at the HSE06 level. In contrast, the PBEsol-optimized structure was used to calculate the phonon dispersion relations as well as the second- and third-order interatomic force constants (FCs). PBEsol was chosen because it provides a good balance between computational accuracy and efficiency, offering a reasonable description of lattice dynamical characteristics.^31^ In addition, the elastic constants, piezoelectric coefficients, ionic dielectric constants, and polar optical phonon frequency were also calculated from the PBEsol-optimized structure. No constraints were imposed on the shape or volume of the unit cell during structural optimization, which continued until the maximum residual force on any atom was less than 0.0001 eV Å^–1^. To minimize Pulay stress,^32^ the energy cutoff was increased to 650 eV (approximately 30% higher than the converged value) for all calculations in which the cell volume was allowed to change. The electronic band structure and DoS were analyzed using the Sumo^33^ package, and the effective carrier masses obtained by parabolic fitting of the band edges were used only for qualitative comparison and discussion, rather than as input parameters for the electronic transport calculations.

To obtain the Seebeck coefficient, electrical conductivity, and electronic thermal conductivity, the electron Boltzmann transport equation was solved using AMSET.^34^ AMSET employs the momentum relaxation time approximation (MRTA) instead of the constant relaxation time approximation (CRTA) to calculate the scattering rates of each electronic state at various temperatures and carrier concentrations, thereby avoiding the large mean absolute percentage errors in carrier mobility that can arise under CRTA.^34,35^ Specifically, AMSET can account for scattering processes arising from acoustic deformation potential (ADP), ionized impurity (IMP), piezoelectric interaction (PIE), and polar optical phonon (POP) scattering. In this study, all four scattering mechanisms—ADP, IMP, POP, and PIE—were considered. The input parameters required by AMSET include deformation potentials, wave function coefficients, and high-frequency dielectric constants obtained from HSE06 functional calculations; other required parameters, including the elastic constants, piezoelectric coefficients, ionic dielectric constants, and polar optical phonon frequencies, were calculated using finite-difference and density functional perturbation theory (DFPT) based on the GGA-PBEsol functional. The static dielectric constant used in the calculations was obtained by summing the high-frequency and ionic dielectric constants. All relevant data are provided in Section 2 of the Supporting Information and in the links listed in the Data Availability section. The convergence test for the interpolation meshes used in the electronic transport calculations can be found in Section 3 of the Supporting Information. In this work, an 85 × 33 × 53 interpolation mesh was used for SrZn_2_S_2_O.

To obtain the second- and third-order FCs required for calculating the lattice thermal conductivity, the finite displacement method as implemented in the Phonopy^36^ and Phono3py^37^ packages was employed. The second-order FCs were computed using a 5 × 2 × 3 supercell (containing 360 atoms) based on the unit cell, while the third-order FCs were evaluated using a smaller 4 × 2 × 2 supercell (containing 256 atoms). We tested the phonon dispersions using different supercell meshes and verified the convergence of the phonon dispersion of SrZn_2_S_2_O with respect to the supercell mesh used for calculating the second-order FCs (Section 4 of the Supporting Information). A total of 21,918 displaced configurations were evaluated in the calculation of the third-order FCs, with a default displacement amplitude of 0.03 Å. The nonanalytical correction (NAC) was also included in the calculations to account for the long-range interactions between ionic charges and macroscopic electric fields. Within the single-mode relaxation time approximation (SMRTA), the lattice thermal conductivity was obtained by solving the phonon Boltzmann transport equation. The convergence of the lattice thermal conductivity with respect to the *q*-point sampling mesh was carefully verified, and a 30 × 30 × 30 mesh was subsequently adopted (Figure S4).

## Results and Discussion

### Crystal Structure

SrZn_2_S_2_O crystallizes in the *Pmn*2_1_ space group (No. 31), with its conventional unit cell identical to the primitive unit cell. The crystal structure is shown in Figure 1a. This compound can be regarded as composed of [Zn_2_S_2_O]^2–^ layers formed by the connection of ZnS_3_O tetrahedra through shared S and O atoms, which are alternately separated by Sr^2+^ cations (Figure 1b). Such a layered mixed-anion framework implies anisotropic transport properties of the material. The Zn atoms are located in tetrahedral coordination environments (Figure 1c), whereas the Sr atoms occupy larger coordination polyhedra (Figure 1d). Due to the preferential orientation of the ZnS_3_O tetrahedra, the overall structure exhibits a noncentrosymmetric polar character, which also reflects the low symmetry of this space group.

**Figure 1:**
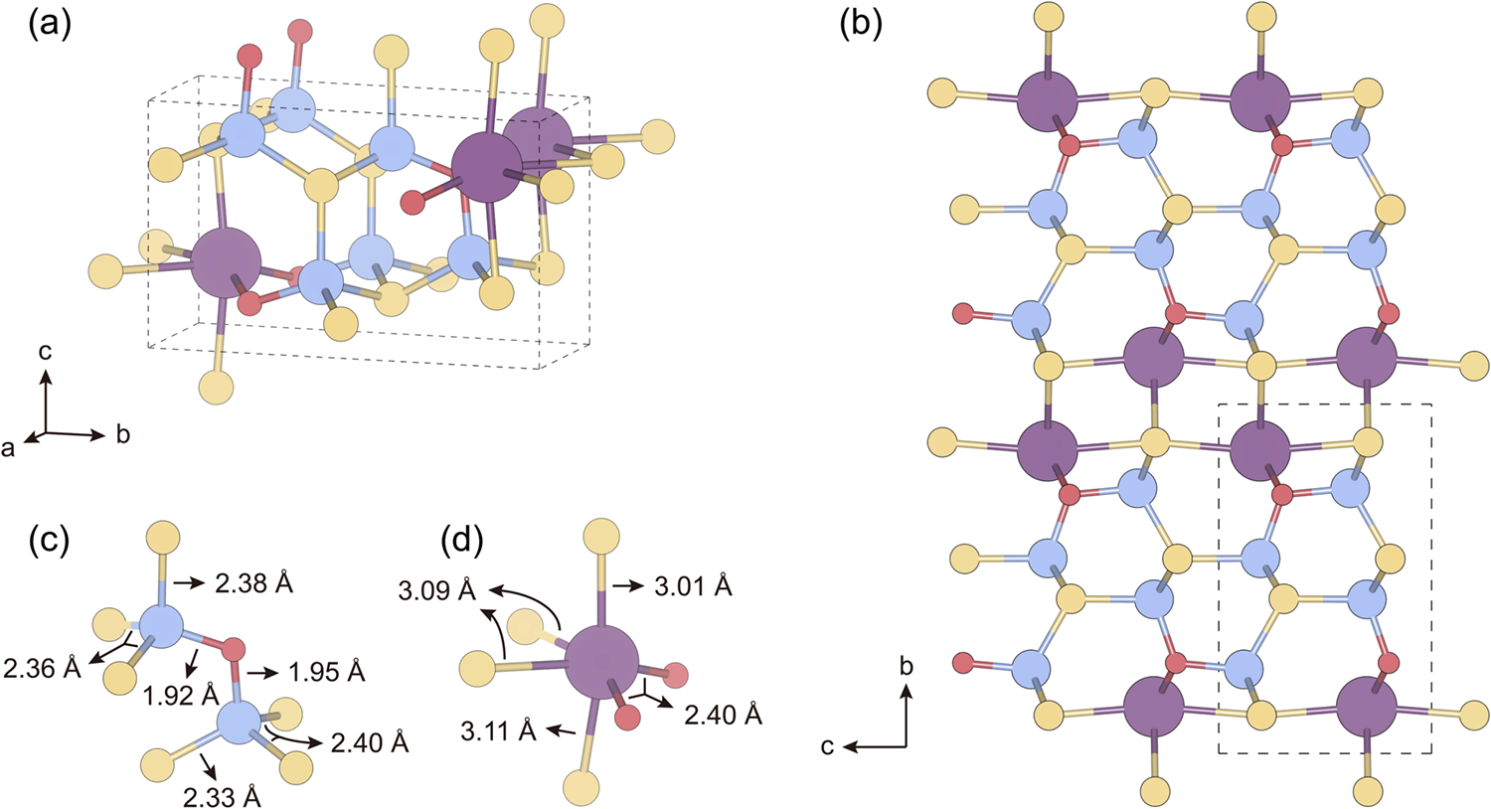
(a) Crystal structure of SrZn_2_S_2_O. The atoms are colored as follows: Sr—purple, Zn—blue, S—yellow, and O—red. This image was generated using the VESTA^38^ software. (b) Side view of the supercell of SrZn_2_S_2_O containing 96 atoms. A unit cell is shown within the dashed line. (c) Two types of ZnS_3_O tetrahedra and the bond lengths within them (bond length data from ref 39). (d) SrS_4_O_2_ polyhedron and the bond lengths within it (bond length data from ref 39).

Table 1 lists the lattice parameters of SrZn_2_S_2_O. The calculated values of SrZn_2_S_2_O are in very good agreement with the experimental ones: PBEsol tends to slightly underestimate the lattice parameters, whereas HSE06 shows the opposite trend. Table 2 presents the interatomic distances of this compound.

**Table 1:** Calculated Lattice Parameters of SrZn_2_S_2_O, with Percentage Differences Relative to Experiment Given in Parentheses

	*a* (Å)	*b* (Å)	*c* (Å)
Tsujimoto et al.^39^ (expt.)	3.87	9.98	6.09
PBEsol	3.85 (−0.63%)	9.93 (−0.55%)	6.05 (−0.68%)
HSE06	3.88 (0.14%)	10.05 (0.65%)	6.11 (0.30%)

**Table 2:** Calculated Interatomic Distances (Å) of SrZn_2_S_2_O, with Percentage Differences Relative to Experiment Given in Parentheses

bonds	expt.^39^	PBEsol	HSE06
Zn(1)–O	1.92	1.92 (0.00%)	1.93 (0.52%)
Zn(1)–S(1)×2	2.36	2.33 (−1.27%)	2.36 (0.00%)
Zn(1)–S(2)	2.38	2.35 (−1.26%)	2.38 (0.00%)
Zn(2)–O	1.95	1.95 (0.00%)	1.96 (0.51%)
Zn(2)–S(1)×2	2.40	2.37 (−1.25%)	2.40 (0.00%)
Zn(2)–S(2)	2.33	2.31 (−0.86%)	2.33 (0.00%)
Sr–O×2	2.40	2.38 (−0.83%)	2.40 (0.00%)
Sr–S(1)	3.01	2.99 (−0.66%)	3.03 (0.66%)
Sr–S(1)	3.11	3.08 (−0.96%)	3.10 (−0.32%)
Sr–S(1)×2	3.09	3.08 (−0.32%)	3.12 (0.97%)

### Electronic Structure

Figure 2 shows the electronic band structure and DoS of SrZn_2_S_2_O calculated by using the HSE06 functional. SrZn_2_S_2_O is a direct-gap semiconductor with a band gap of 3.52 eV. Experimentally, the optical band gap of SrZn_2_S_2_O was determined to be 3.86 eV from UV–vis–NIR diffuse reflectance measurements,^39^ whereas the calculated optical band gap in this work based on the HSE06 functional is 3.62 eV. The difference between the two values is merely 0.24 eV, indicating good overall agreement. Such a deviation is typical for hybrid functional predictions of wide-band gap materials. Both the valence band maximum (VBM) and conduction band minimum (CBM) are located at the Γ point. The valence bands near the VBM are mainly dominated by S 3p states, while the conduction bands near the CBM are primarily derived from Zn 4s states, with a non-negligible contribution from S 3s states. Owing to the strong spatial delocalization of the Zn 4s orbitals, the CBM exhibits pronounced dispersion, indicating a small electron effective mass.

**Figure 2:**
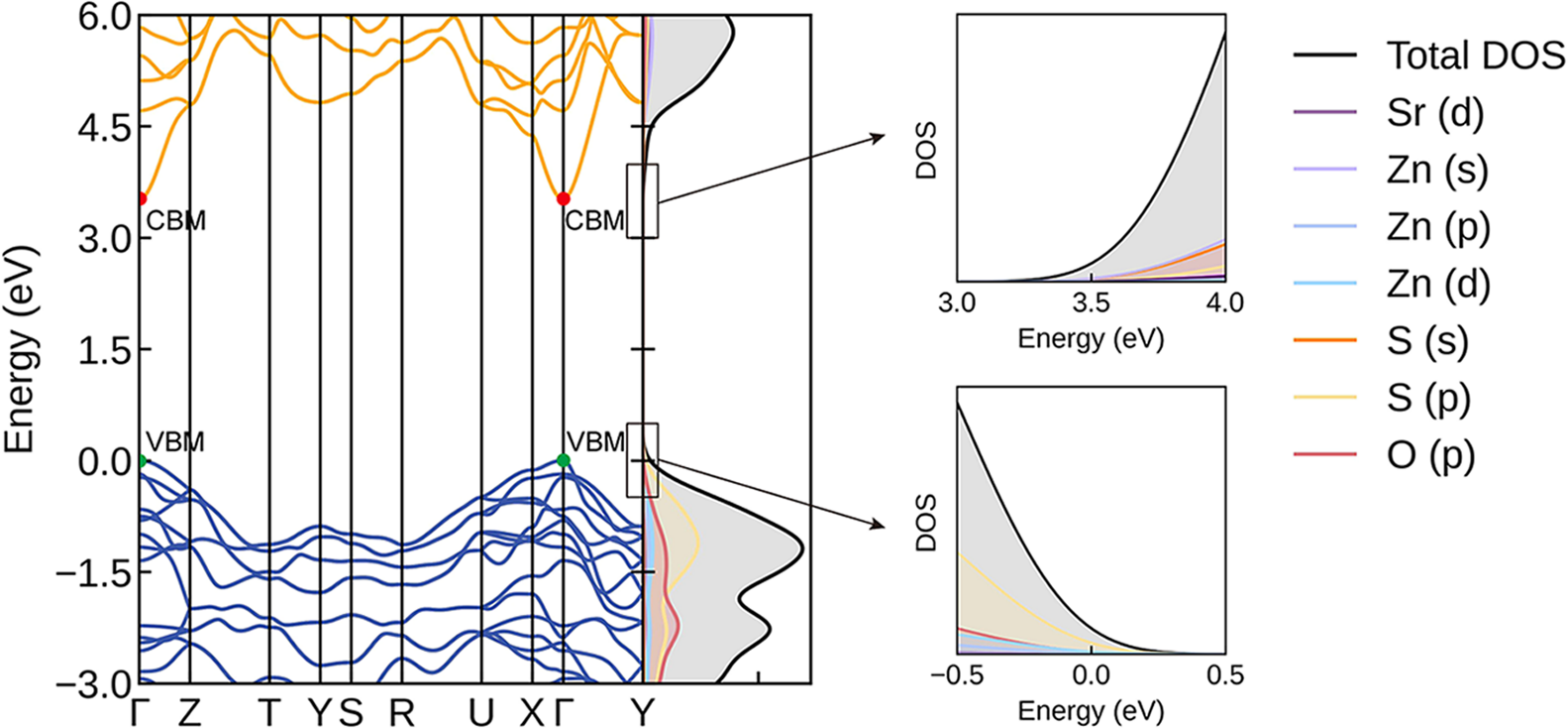
Electronic band structure and DoS of SrZn_2_S_2_O calculated using the HSE06 functional. The band structure is plotted along the Bradley-Cracknell *k*-point path.^40^ The conduction and valence bands are colored in orange and blue, respectively. The two side panels display the DoS near the CBM (upper) and VBM (lower). All plots were generated using the Sumo^33^ package.

The effective masses of carriers at the band edges are given in Table 3. A larger band curvature corresponds to a smaller effective mass. The conduction bands of SrZn_2_S_2_O exhibit high curvature along the Γ–*X*, Γ–*Y*, and Γ–*Z* directions, resulting in relatively small electron effective masses. This favors high carrier mobility and consequently enhances n-type electrical conductivity. This result is consistent with the previous analysis showing that the strong spatial delocalization of the Zn 4s orbitals leads to a highly dispersive CBM. Moreover, the similar curvatures along the three directions indicate nearly isotropic electron effective masses. In contrast, for holes, the valence bands along the Γ–*X* and Γ–*Z* directions are flatter than those along Γ–*Y*, leading to significantly larger hole effective masses and pronounced anisotropy. The larger hole effective masses along Γ–*X* and Γ–*Z* imply limited hole mobility, which is unfavorable for p-type transport.

**Table 3:** Effective Masses of Carriers in SrZn_2_S_2_O Calculated Using Sumo^33^

high-symmetry path	hole effective masses	electron effective masses
Γ–*X*	1.53	0.27
Γ–*Y*	0.22	0.22
Γ–*Z*	1.75	0.29

### Electronic Transport Properties

In this section, we analyze the electronic transport properties of SrZn_2_S_2_O. The calculations were carried out using the AMSET package. The temperature range was set from 100 to 900 K, as thermogravimetric (TG) analysis indicates that SrZn_2_S_2_O exhibits excellent thermal stability in air up to 923 K.^39^

Figure 3 presents the electronic transport properties and average scattering rates of SrZn_2_S_2_O. The electrical conductivity decreases with increasing temperature (Figure 3a,h), which can be attributed to the increase in the total scattering rate with increasing temperature (Figure 3e,l), leading to shorter carrier lifetimes and thus lower electrical conductivity. Conversely, the electrical conductivity increases with carrier concentration due to their direct positive correlation. Furthermore, at high carrier concentrations, the temperature dependence of electrical conductivity becomes weaker. This is because, at low carrier concentrations, POP scattering dominates and is strongly temperature-dependent, as shown in Figure 3e,f,l,m, whereas, at high carrier concentrations, IMP scattering becomes the dominant mechanism and its scattering rate is insensitive to temperature. Under the conditions corresponding to the maximum *ZT* value, IMP scattering exhibits the highest scattering rate near the band edges, while POP scattering becomes more significant at higher carrier energies (Figure 3g,n). Overall, POP and IMP scatterings are the primary mechanisms governing the electrical conductivity of SrZn_2_S_2_O.

**Figure 3:**
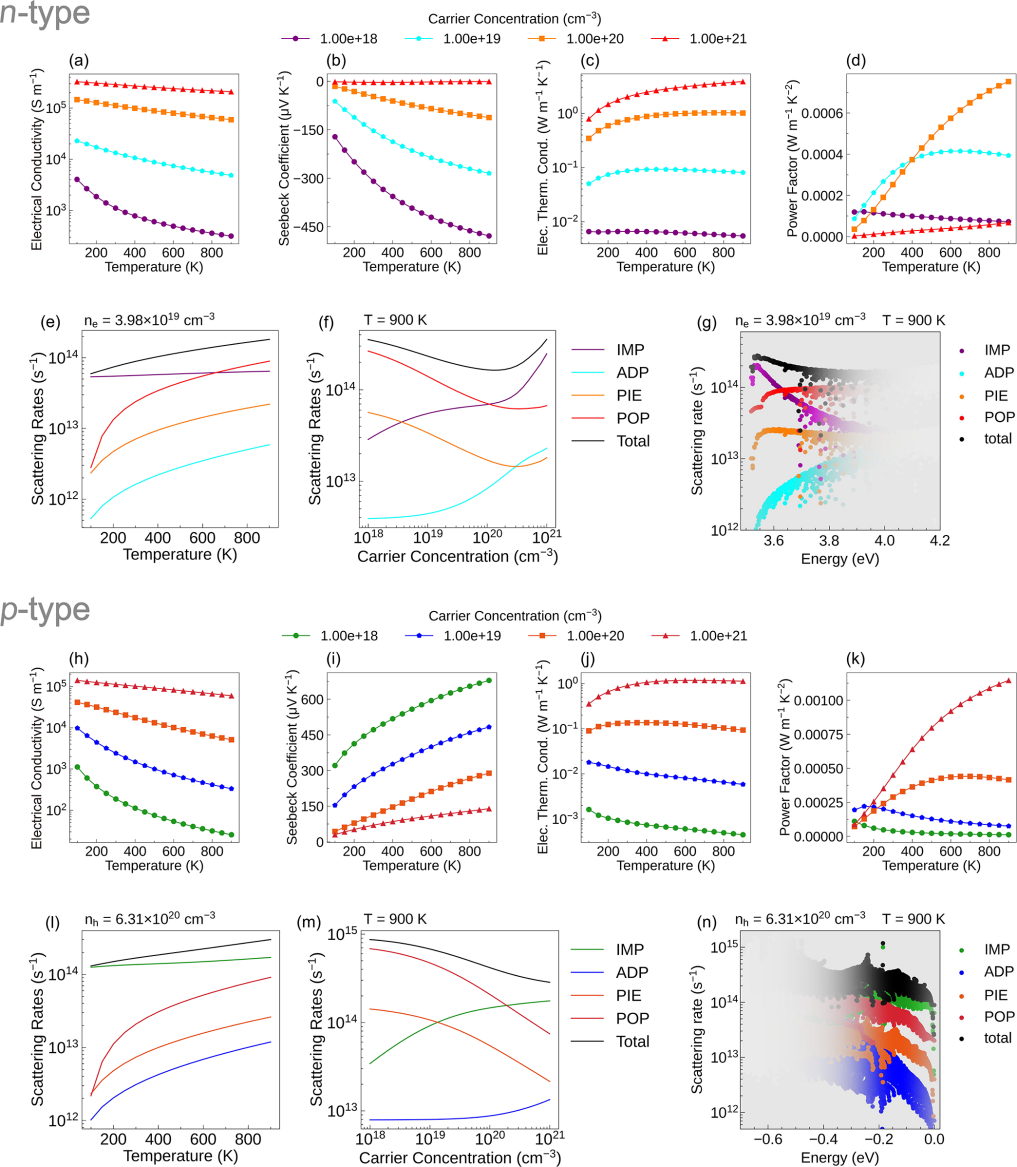
Calculated electronic transport properties and average scattering rates of SrZn_2_S_2_O. Panels (a–d) and (h–k) show the temperature-dependent n-type and p-type electronic transport properties, respectively, at four different carrier concentrations. Panels (e–g) and (l–n) present the n-type and p-type average scattering rates evaluated under the carrier concentrations and the temperature corresponding to the predicted maximum *ZT* values. The average scattering rates are plotted as functions of (e, l) temperature, (f, m) carrier concentration, and energy relative to (g) the CBM and (n) the VBM. In panels (g, n), the color intensity indicates the availability of carrier-scattering channels across the bands and their weighted contributions to the total mobility, as determined by the derivative of the Fermi–Dirac distribution function.

To contextualize the electronic transport properties of SrZn_2_S_2_O, we compared our results with those of BiCuSeO, another mixed-anion oxide. BiCuSeO is widely regarded as a promising TE material owing to its ultralow intrinsic thermal conductivity.^41^ Section 6 of the Supporting Information lists the calculated n-type and p-type electrical conductivities and Seebeck coefficients of SrZn_2_S_2_O and BiCuSeO at 300, 600, and 900 K. At a carrier concentration of 1 × 10^20^ cm^–3^, the n-type electrical conductivities of SrZn_2_S_2_O are 1.12 × 10^5^, 7.95 × 10^4^, and 5.93 × 10^4^ S m^–1^ at 300, 600, and 900 K, respectively, whereas the corresponding values for BiCuSeO are approximately 3.88 × 10^4^, 2.17 × 10^4^, and 1.31 × 10^4^ S m^–1^.^42^ Obviously, SrZn_2_S_2_O exhibits a higher n-type electrical conductivity at all three temperatures. Moreover, as shown in Table S1, even at a more experimentally accessible carrier concentration of *n* = 1 × 10^19^ cm^–3^, SrZn_2_S_2_O maintains higher electrical conductivity than BiCuSeO at the three temperatures. For p-type electrical conductivity, SrZn_2_S_2_O also shows higher values than BiCuSeO at the same temperatures and carrier concentrations.

The Seebeck coefficient increases with an increasing temperature but decreases with an increasing carrier concentration, which is the opposite of the trend observed for the electrical conductivity. This behavior arises from the intrinsic trade-off between the Seebeck coefficient and the electrical conductivity. At a carrier concentration of 1 × 10^20^ cm^–3^, the n-type Seebeck coefficients of SrZn_2_S_2_O are 47, 84.5, and 112 μV K^–1^ at 300, 600, and 900 K, respectively, while those of BiCuSeO are approximately 212, 237, and 256 μV K^–1^ at the same temperatures. SrZn_2_S_2_O exhibits smaller Seebeck coefficients than BiCuSeO, and this trend persists at a lower carrier concentration (1 × 10^19^ cm^–3^) (Table S1). A comparison of p-type Seebeck coefficients at the same temperatures and carrier concentrations reveals that BiCuSeO exhibits higher values (Tables S3 and S4). Therefore, SrZn_2_S_2_O has a higher electrical conductivity, whereas BiCuSeO possesses larger Seebeck coefficients. However, achieving a high power factor requires a balance between the electrical conductivity and the Seebeck coefficient. The maximum *n*-type power factor of SrZn_2_S_2_O reaches 753 μW m^–1^ K^–2^ within the carrier concentration range of 10^18^–10^21^ cm^–3^, while BiCuSeO exhibits a much higher maximum n-type power factor of about 1700 μW m^–1^ K^–2^ within the range of 10^19^–10^22^ cm^–3^. Evidently, BiCuSeO achieves a better balance between the electrical conductivity and the Seebeck coefficient in n-type transport. On the other hand, the maximum *p*-type power factor of SrZn_2_S_2_O is 1150 μW m^–1^ K^–2^, which exceeds that of BiCuSeO (≈ 770 μW m^–1^ K^–2^), indicating that SrZn_2_S_2_O possesses potential advantages in p-type transport performance.

### Phonon Transport Properties

No imaginary frequencies are observed in the calculated phonon dispersion of SrZn_2_S_2_O (Figure 4a), indicating that the system is dynamically stable. Overall, the phonon branches of SrZn_2_S_2_O are relatively flat, suggesting low phonon group velocities, as the group velocity is determined by the slope of the phonon branches. The atom-projected phonon DoS reveals that, in the low-frequency region below 5 THz, the phonon modes are mainly derived from the vibrations of Zn atoms, with a non-negligible contribution from Sr atoms. In the mid-frequency range 5–10 THz, the vibrations are dominated by S atoms, while the contributions from Sr and Zn atoms are relatively small. In the high-frequency range 10–17 THz, the phonon modes are primarily governed by the vibrations of the O atoms. The presence of Sr and Zn atoms shifts part of the optical branches toward lower frequencies, bringing them closer to the acoustic branches and thereby providing more scattering channels for acoustic-optical phonon interactions. Moreover, avoided crossings between the acoustic and optical phonon branches can be observed in the phonon dispersion (Figure 4b). These avoided crossings arise from phonon-mode hybridization, which locally flattens the acoustic branches near the crossing points, thereby reducing the slope of their dispersion curves and lowering the group velocities of the acoustic phonons.^43^ Although the optical branches involved in the avoided crossings acquire larger dispersion slopes and exhibit higher group velocities, phonon thermal transport is dominated by long-wavelength acoustic phonons along the Γ–*X*, Γ–*Y*, and Γ–*Z* directions. Therefore, we highlight the representative avoided crossings between the acoustic and low-frequency optical branches in the low-frequency region along these directions as they play the most significant role in suppressing the lattice thermal conductivity.

**Figure 4:**
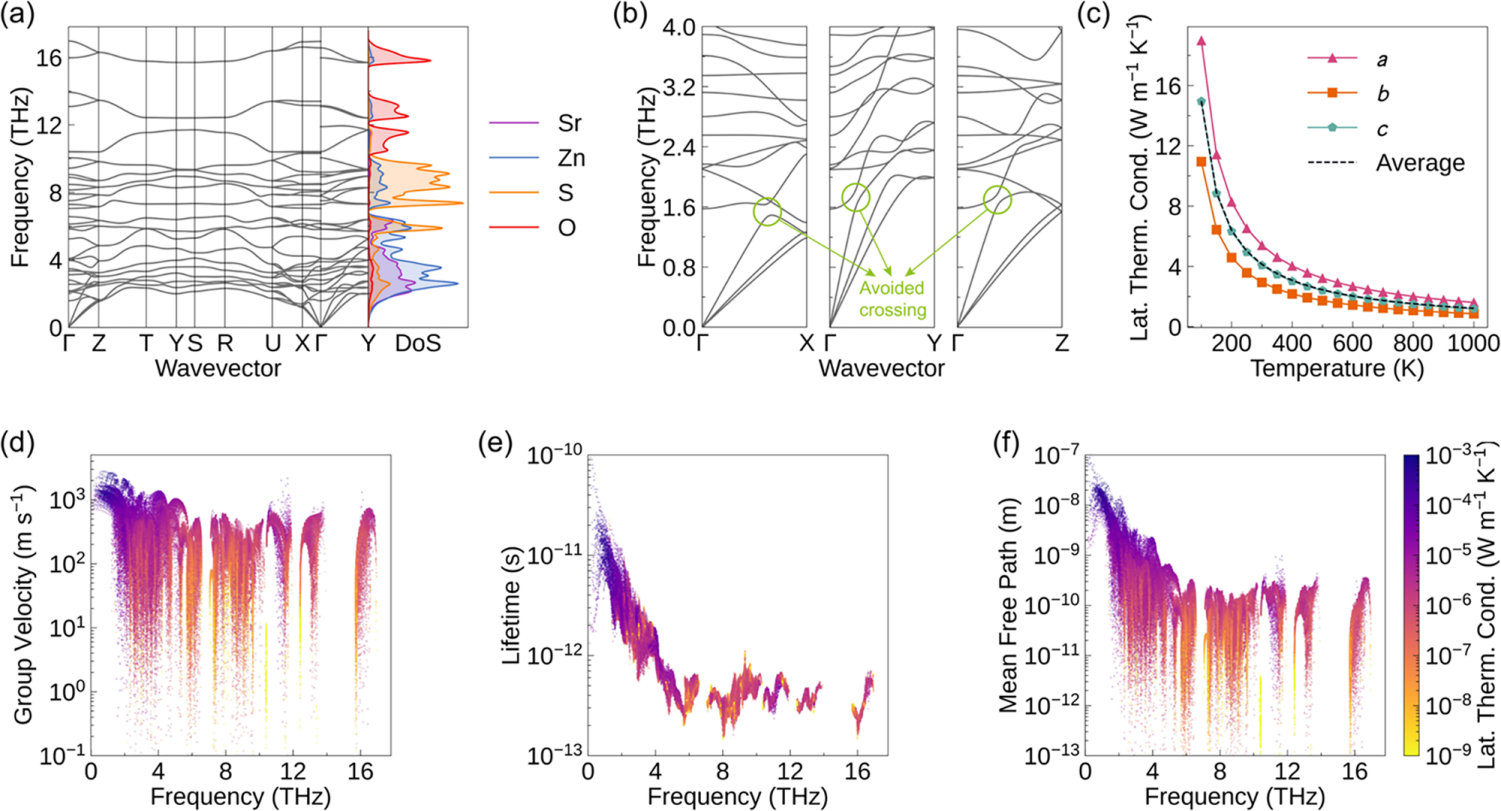
(a) Phonon dispersion and atom-projected phonon DoS of SrZn_2_S_2_O with NAC applied. The phonon dispersion was plotted using ThermoParser,^52^ and the high-symmetry path was constructed following the Bradley-Cracknell scheme.^40^ (b) Enlarged phonon dispersions in the low-frequency region along the Γ–*X*, Γ–*Y*, and Γ–*Z* directions. The green circles highlight the avoided crossings between the acoustic and optical branches. (c) Lattice thermal conductivity of SrZn_2_S_2_O along the *a*, *b*, and *c* directions and their average, as a function of temperature. (d) Phonon group velocity, (e) phonon lifetime at 900 K, and (f) phonon mean free path at 900 K, all plotted as functions of frequency. The color bar represents the projected contribution to the lattice thermal conductivity at 900 K, with yellow and dark blue corresponding to low and high contributions, respectively.

Figure 4b shows the variation of the lattice thermal conductivity of SrZn_2_S_2_O with temperature. The lattice thermal conductivity was calculated within the SMRTA, considering only three-phonon scattering mechanisms while neglecting higher-order phonon–phonon and phonon-defect scattering processes. Moreover, since the RTA treats normal scattering processes as resistive ones, it tends to underestimate the lattice thermal conductivity.^44,45^ However, this underestimation partly compensates for the absence of other scattering mechanisms, resulting in good overall agreement between calculated and experimental values.^41,42,46,47^ SrZn_2_S_2_O exhibits a relatively low lattice thermal conductivity, with an average value of 1.35 W m^–1^ K^–1^ at 900 K, which is, however, significantly higher than that of BiCuSeO (0.36 W m^–1^ K^–1^) at the same temperature. This low lattice thermal conductivity can be explained from the perspectives of the phonon group velocity and the phonon lifetime.

As shown in Figure 4c, the phonon group velocities of SrZn_2_S_2_O range from 10^–1^ to 3 × 10^3^ m s^–1^, with an average value of 341 m s^–1^, which is significantly lower than that of BiCuSeO (2107 m s^–1^).^47^ The low phonon group velocity is closely related to the large atomic mass of Sr, which suppresses phonon propagation. In addition, the low lattice thermal conductivity of SrZn_2_S_2_O is related to the avoided crossings between the acoustic and optical branches in the phonon dispersion. These avoided crossings suppress phonon transport by reducing the group velocities of long-wavelength acoustic phonons that make the dominant contribution to the lattice thermal conductivity.

The phonon lifetime is jointly influenced by the phonon scattering intensity and the number of available scattering channels. The Grüneisen parameter is commonly used to quantify the anharmonicity of a material: a larger value indicates stronger anharmonicity and thus more intense phonon–phonon scattering processes.^48,49^ The Grüneisen parameter of SrZn_2_S_2_O was calculated using the following equation

2 $$\gamma_{\mathrm{rms}}=\sqrt{\frac{\sum_{\lambda }C_{\lambda }\gamma_{\lambda }^{2}}{\sum_{\lambda }C_{\lambda }}}$$

where γ_rms_ denotes the heat-capacity-weighted root-mean-square (rms) Grüneisen parameter, *C*_λ_ is the mode-dependent heat capacity, and γ_λ_ is the mode Grüneisen parameter. Since the Umklapp scattering rate is proportional to the square of the Grüneisen parameter, γ_λ_ was squared in the calculation.^50,51^ In the computation of γ_rms_, the mode heat capacities *C*_λ_ at 300 K were used. As shown in Figure S5, the γ_rms_ value was calculated only within the range of phonon modes that contribute up to 90% of the total lattice thermal conductivity, ensuring that the result reflects the anharmonicity of the phonons that primarily governs heat transport. The Grüneisen parameter of SrZn_2_S_2_O is 0.46, which is significantly smaller than that of the low-thermal-conductivity material BiCuSeO (1.5).^47^ Therefore, the relatively short phonon lifetimes in SrZn_2_S_2_O cannot be primarily ascribed to strong phonon–phonon scattering. On the other hand, the presence of Sr and Zn atoms shifts some optical modes toward lower frequencies, thereby opening more phonon–phonon scattering channels. This explains the pronounced decrease in the phonon lifetimes in the low-frequency region (Figure 4e). In addition, the low-symmetry *Pmn*2_1_ crystal structure lifts phonon-mode degeneracy and introduces more inequivalent vibrational modes, further increasing the number of allowed phonon scattering channels, which enhances phonon scattering and consequently suppresses the lattice thermal conductivity.

Figure 4f shows the variation of the phonon mean free path (MFP) of SrZn_2_S_2_O as a function of frequency. Low-frequency phonons exhibit relatively long mean free paths due to their higher group velocities and longer phonon lifetimes. A large number of phonon modes with significant contributions to the lattice thermal conductivity can be observed around 1 × 10^–8^ m. This indicates that when nanostructures with characteristic sizes of *L* ≲ 10^–8^ m (10 nm) are introduced, these long-wavelength phonons will experience pronounced boundary scattering, thereby effectively reducing the lattice thermal conductivity. Therefore, nanostructuring is a feasible and effective strategy for further lowering the lattice thermal conductivity of SrZn_2_S_2_O.

In addition, we observed that SrZn_2_S_2_O exhibits the lowest lattice thermal conductivity along the *b* direction, which is mainly attributed to the bond heterogeneity in this direction. The structure of SrZn_2_S_2_O can be described as alternating stacking of ZnS_3_O layers and SrS_4_O_2_ layers. Along the *b* direction, phonon transport necessarily traverses these alternately arranged layers, and distinct bonding characteristics may exist between these different layered units. To quantitatively evaluate the bond strength, we calculated the negative integrated crystal orbital Hamilton population (–ICOHP) for each type of chemical bond, as listed in Table 4. This parameter is an effective indicator of the bond strength. As shown in Table 4, there are noticeable differences in the bond strengths within the ZnS_3_O layers and those within the SrS_4_O_2_ layers. This bonding heterogeneity introduces clear discontinuities in bonding characteristics along the *b* direction. Because phonons propagating along this direction must cross layered units with distinct bonding features, such bonding differences disrupt the continuous propagation of phonons, thereby enhancing phonon scattering and suppressing phonon heat transport. In this way, the bonding heterogeneity provides a structural origin for suppressed phonon transport along the *b* direction. It should be emphasized that, in the background discussion, we mentioned that the introduction of additional anions may contribute to a reduction in lattice thermal conductivity by enhancing phonon scattering. This expectation arises from the mixed-anion strategy, which may introduce chemical bonds with different strengths into the system, thereby leading to bonding heterogeneity.^23,24^ In such cases, relatively weaker bonds can lower the vibrational frequencies of lighter atoms and increase the likelihood of scattering between acoustic and optical phonons. However, although SrZn_2_S_2_O is a mixed-anion compound from a compositional perspective, the –ICOHP analysis reveals no pronounced differences in the bonding strengths between different anions coordinated to the same cation. This indicates that the bonding heterogeneity in the system does not primarily originate from mixed-anion characteristics. Therefore, in the case of SrZn_2_S_2_O, the mixed-anion nature does not play a decisive role in determining the lattice thermal conductivity.

**Table 4:** –ICOHP Values per Bond for SrZn_2_S_2_O

bonds	–ICOHP (eV)
Zn(1)–O	1.33
Zn(1)–S(1)×2	1.33
Zn(1)–S(2)	1.21
Zn(2)–O	1.24
Zn(2)–S(1)×2	1.18
Zn(2)–S(2)	1.41
Sr–O×2	0.62
Sr–S(1)	0.61
Sr–S(1)	0.53
Sr–S(1)×2	0.46

### TE Figure of Merit

Finally, based on eq 1, the *ZT* values were calculated by combining the calculated electronic transport properties and lattice thermal conductivity using the ThermoParser^52^ software. The total thermal conductivity of the system was obtained by summing the lattice and electronic contributions. The carrier concentration was varied from 10^18^–10^22^ cm^–3^, and the temperature ranged from 100 to 900 K. Figure 5a,c presents the heat maps of the predicted average n-type and p-type *ZT* values as functions of temperature and carrier concentration. Tables 5 and 6 list, for the n-type and p-type cases, the maximum *ZT* values along each crystallographic direction, the averaged maximum *ZT* values, and the corresponding carrier concentrations, lattice thermal conductivities, power factors, and electronic thermal conductivities. For n-type transport, the maximum average *ZT* reaches 0.38, with the highest value of 0.48 along the *b* direction, owing to its lower thermal conductivity (Table 5). For p-type transport, the maximum average *ZT* is 0.43, while the *c* direction exhibits a higher value of 0.55 (Table 6). Although the thermal conductivity is lower along the *b* direction, the p-type power factor along this direction is very low under the carrier concentration and temperature conditions corresponding to the maximum *ZT*. In contrast, the *c* direction exhibits a power factor as high as 1250 μW m^–1^ K^–2^ under the same conditions. Therefore, the higher p-type *ZT* along the *c* direction primarily arises from the superior power factor.

**Figure 5:**
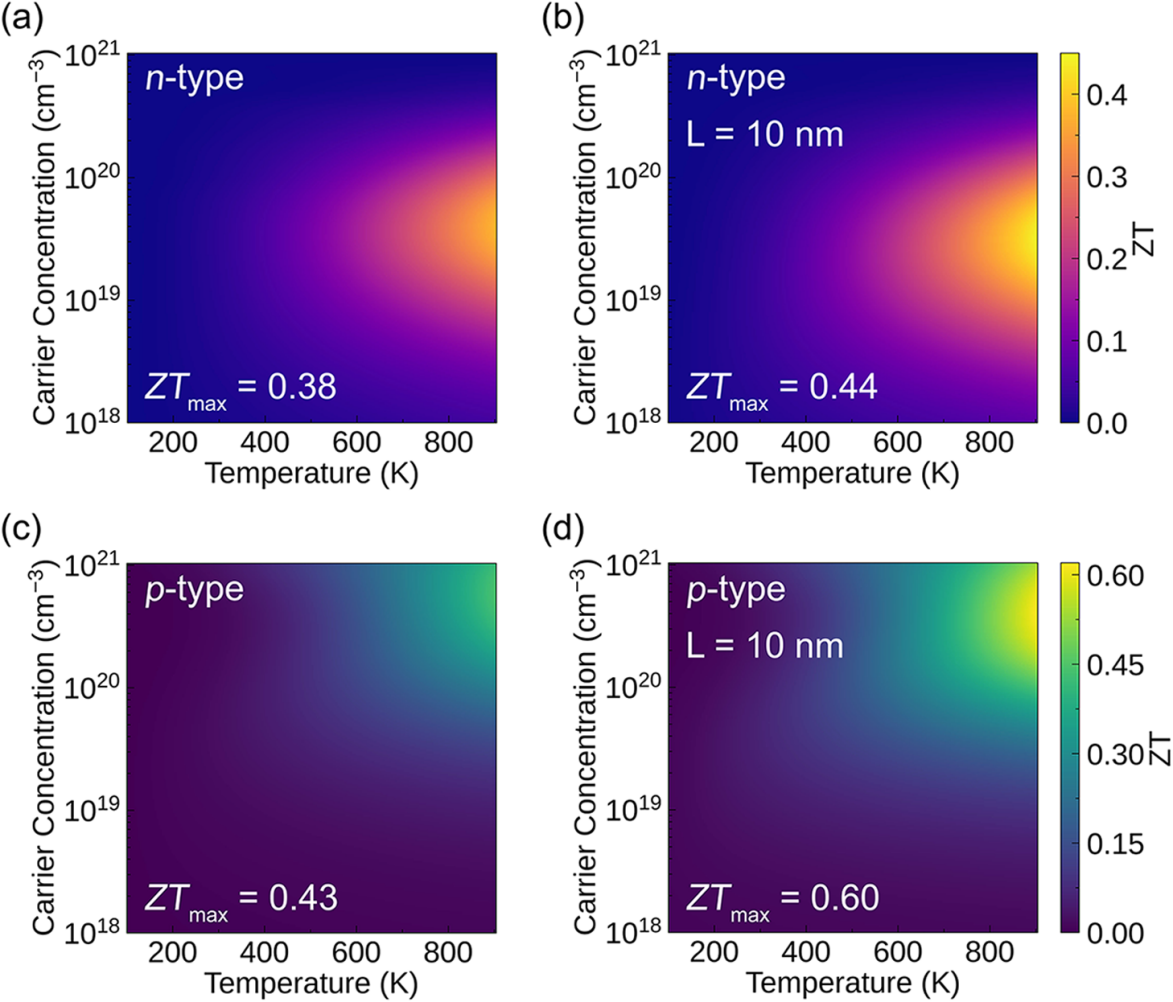
Heat maps of the average *ZT* values of SrZn_2_S_2_O as functions of temperature and carrier concentration. (a) n-type and (c) p-type results for the intrinsic system, and (b) n-type and (d) p-type results for the nanostructured system (*L* = 10 nm). The analysis was conducted using ThermoParser.^52^

**Table 5:** Predicted Maximum n-Type *ZT* Values of Intrinsic and Nanostructured SrZn_2_S_2_O at 900 K, Including Direction-Resolved Maximum Values and the Maximum Average *ZT* Value, Together with the Corresponding Carrier Concentration (*n*), Lattice Thermal Conductivity (κ_*l*_), Power Factor (PF), and Electronic Thermal Conductivity (κ_e_)

MFP (nm)	direction	*n* (cm^–3^)	max *ZT*	κ_l_ (W m^–1^ K^–1^)	PF (μW m^–1^ K^–2^)	κ_e_ (W m^–1^ K^–1^)
-	*a*	–5.01 × 10^19^	0.34	1.78	888	0.59
	*b*	–3.98 × 10^19^	0.48	0.95	703	0.36
	*c*	–3.16 × 10^19^	0.36	1.32	636	0.28
	average	–3.98 × 10^19^	0.38	1.35	737	0.38
10	*a*	–3.16 × 10^19^	0.36	1.07	536	0.26
	*b*	–2.55 × 10^19^	0.65	0.41	418	0.17
	*c*	–3.16 × 10^19^	0.42	0.71	432	0.21
	average	–3.16 × 10^19^	0.44	0.73	472	0.23

**Table 6:** Predicted Maximum p-Type *ZT* Values of Intrinsic and Nanostructured SrZn_2_S_2_O at 900 K, Including Direction-Resolved Maximum Values and the Maximum Average *ZT* Value, together with the Corresponding Carrier Concentration (*n*), Lattice Thermal Conductivity (κ_l_), Power Factor (PF), and Electronic Thermal Conductivity (κ_e_)

MFP (nm)	direction	*n* (cm^–3^)	max *ZT*	κ_l_ (W m^–1^ K^–1^)	PF (μW m^–1^ K^–2^)	κ_e_ (W m^–1^ K^–1^)
-	*a*	3.98 × 10^20^	0.45	1.78	1340	0.88
	*b*	1.00 × 10^21^	0.25	0.95	352	0.34
	*c*	7.94 × 10^20^	0.55	1.32	1250	0.71
	average	6.31 × 10^20^	0.43	1.35	1000	0.74
10	*a*	2.51 × 10^20^	0.61	1.07	1050	0.49
	*b*	7.94 × 10^20^	0.38	0.41	285	0.26
	*c*	5.01 × 10^20^	0.77	0.71	970	0.42
	average	5.01 × 10^20^	0.60	0.73	847	0.54

### Nanostructuring

Nanostructuring can reduce the lattice thermal conductivity of materials by enhancing phonon scattering and shortening the phonon mean free path. Figure 5b,d shows the heat maps of the average n-type and p-type *ZT* values of SrZn_2_S_2_O as functions of temperature and carrier concentration after nanostructuring. The complete heat maps of the n-type and p-type *ZT* along different crystallographic directions before and after nanostructuring, as functions of temperature and carrier concentration, are provided in Section 8 of the Supporting Information. By comparing the intrinsic and nanostructured lattice thermal conductivities listed in Tables 5 and 6, it can be observed that when the crystal size is reduced to 10 nm, the average lattice thermal conductivity at 900 K decreases by approximately 46%, with a reduction of more than 50% along the *b* direction. Moreover, this reduction does not take into account the additional phonon scattering that may arise from external doping, which would further suppress the lattice thermal conductivity. Therefore, the nanostructured lattice thermal conductivities presented here should be regarded as upper-limit estimates, and further reductions are expected in practice. Nanostructuring not only affects the thermal conductivity but also influences the power factor. As summarized in Tables 5 and 6, under optimal doping and temperature conditions, nanostructuring results in a decrease of approximately 36% in the average n-type power factor and about 15% in the average p-type power factor. Consequently, the maximum average n-type *ZT* shows no significant improvement after nanostructuring. However, due to the smaller reduction in the p-type power factor, the maximum average p-type *ZT* increases from 0.43 to 0.60, demonstrating the potential advantage of SrZn_2_S_2_O in p-type performance after nanostructuring.

Given that, among the three crystallographic directions, the n-type *ZT* attains its maximum along the *b* direction, whereas the p-type *ZT* is highest along the *c* direction, we further discuss the effect of nanostructuring on the maximum *ZT* along these two favorable crystallographic directions. For n-type performance along the *b* direction, nanostructuring increases the maximum *ZT* from 0.48 to 0.65 (Table 5), indicating that the reduction in thermal conductivity dominates among the competing effects of reduced thermal conductivity and reduced power factor. Along the *c* direction, although the p-type power factor decreases by approximately 22%, this reduction is much smaller than that of the thermal conductivity (which decreases by 44%) (Table 6). Consequently, the maximum p-type *ZT* increases from 0.55 to 0.77. It is worth noting, however, that the maximum p-type *ZT* corresponds to a relatively high carrier concentration; therefore, a well-designed and experimentally feasible doping strategy is essential to achieve this enhancement.

## Conclusions

In summary, the TE properties of SrZn_2_S_2_O were systematically investigated based on DFT calculations. The results reveal that the large atomic mass of Sr reduces the phonon group velocities of the system, while the avoided crossings between acoustic and optical branches weaken the group velocities of long-wavelength acoustic phonons that contribute most significantly to the lattice thermal conductivity. In addition, the presence of Sr and Zn, the structural complexity of SrZn_2_S_2_O, and the bonding heterogeneity enhance the phonon scattering. Under the combined effects of these factors, SrZn_2_S_2_O exhibits low lattice thermal conductivity. At 900 K, the maximum average n-type *ZT* reaches 0.38 (with 0.48 along the *b* direction), while the maximum average p-type *ZT* is 0.43 (with 0.55 along the *c* direction). Compared with the prototypical mixed-anion oxide BiCuSeO, SrZn_2_S_2_O exhibits a higher maximum p-type power factor of up to 1150 μW m^–1^ K^–2^, despite its relatively high thermal conductivity. Further analysis shows that nanostructuring effectively suppresses phonon transport, reducing the lattice thermal conductivity in all directions by 40% or more at a grain size of 10 nm. As a result, the maximum n-type *ZT* along the *b* direction increases to 0.65, and the p-type *ZT* along the *c* direction increases to 0.77. Although nanostructuring can further reduce the thermal conductivity, it also leads to a decrease in the power factor.

In conclusion, SrZn_2_S_2_O demonstrates great potential as a novel high-temperature TE material owing to its relatively high maximum p-type power factor, elemental abundance, and environmental friendliness. However, its relatively high thermal conductivity remains the main limitation for further performance improvement. Therefore, achieving a balance between maintaining a high power factor and reducing the thermal conductivity will be the key to realizing SrZn_2_S_2_O as an efficient TE material. Considering that both BaZnOS and SrZnOS can be structurally stabilized,^53,54^ we propose that introducing a small amount of Ba to substitute Sr in SrZn_2_S_2_O may enhance phonon scattering through mass mismatch and local structural distortion, thereby effectively reducing the lattice thermal conductivity. This strategy may improve the overall TE performance, although the effects of Ba substitution on phase stability, charge-carrier transport, and power factor still require systematic investigation through experimental studies and theoretical calculations.

## Supplementary Material



## Data Availability

The computational input and output data are available in a publicly accessible online repository at https://zenodo.org/records/17709520.

## Notes

Convergence tests for energy cutoffs and *k*-point meshes, AMSET setting, convergence tests of interpolation meshes for electronic transport properties, convergence tests of phonon supercell meshes, convergence tests of lattice thermal conductivity with respect to *q*-point sampling meshes, calculated electrical conductivity and Seebeck coefficient of SrZn_2_S_2_O and BiCuSeO, cumulative lattice thermal conductivity, and anisotropic *ZT* (PDF)
