# Supplementary material for: Computational Investigation of the Thermoelectric Performance of Environmentally Friendly and Earth-Abundant SrZn2S2O
Source: ACS Appl Energy Mater. 2026 Jan 24;9(3):1891–902. doi: 10.1021/acsaem.5c03742 (PMC12892240; doi:10.1021/acsaem.5c03742)
Supplement: Supplementary file 1 [file ae5c03742_si_001.pdf]

Supporting Information

Computational Investigation of the Thermoelectric Performance of Environmentally Friendly and Earth-Abundant $SrZn_2S_2O$

Shipeng Bi[a], *Katarina Brlec*[a], *Alexander G. Squires*[b], *and David O. Scanlon*[b,*]

[a] *Department of Chemistry, University College London, 20 Gordon Street, London WC1H 0AJ, United Kingdom*

[b] *School of Chemistry, University of Birmingham, Edgbaston, Birmingham B15 2TT, United Kingdom*

[*] Corresponding author. E-mail: d.o.scanlon@bham.ac.uk

1 Convergence tests for energy cutoffs and *k*-point meshes

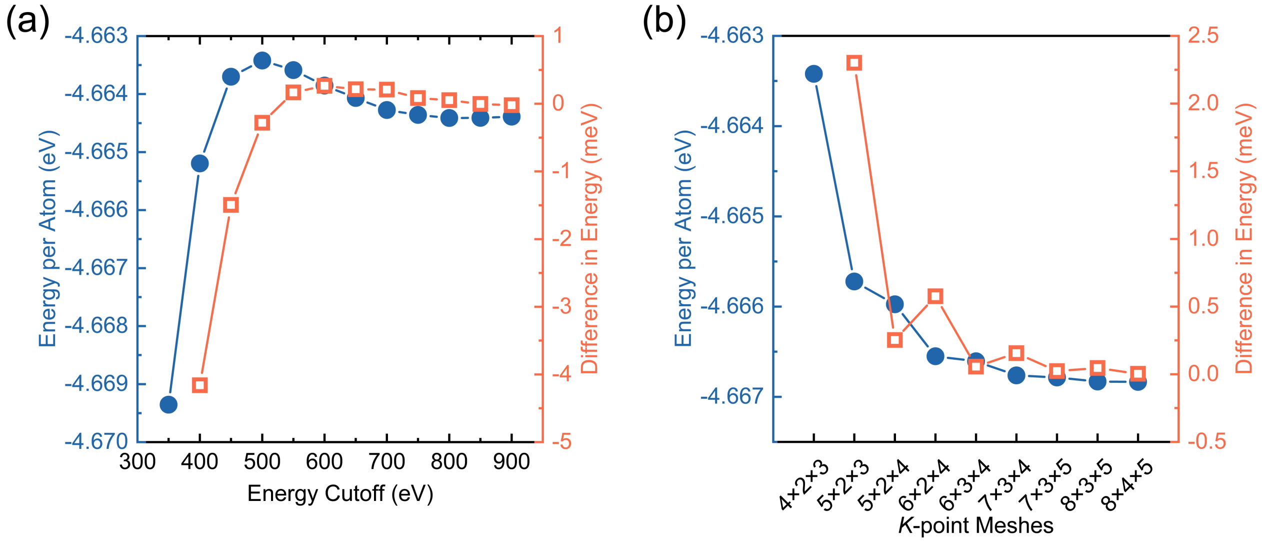

Figure S1. Convergence tests of (a) the energy cutoffs and (b) the *k*-point meshes for the $SrZn_2S_2O$ unit cell. The square symbols represent the average energy per atom, while the circular symbols represent the energy difference between consecutive tests. An energy cutoff of 500 eV and a *k*-point mesh of 7 × 3 × 4 were chosen for the subsequent calculations.

2 AMSET setting

High-frequency dielectric constants = $\begin{bmatrix} 4.46 & 0 & 0 \\ 0 & 4.13 & 0 \\ 0 & 0 & 4.13 \end{bmatrix}$

Static dielectric constants = $\begin{bmatrix} 9.88 & 0 & 0 \\ 0 & 8.71 & 0 \\ 0 & 0 & 8.78 \end{bmatrix}$

Elastic constants (GPa) = $\begin{bmatrix} 126.1 & 54.7 & 34.9 & 0 & 0 & 0 \\ 54.7 & 107.6 & 41.9 & 0 & 0 & 0 \\ 34.9 & 41.9 & 128.5 & 0 & 0 & 0 \\ 0 & 0 & 0 & 26.6 & 0 & 0 \\ 0 & 0 & 0 & 0 & 20.3 & 0 \\ 0 & 0 & 0 & 0 & 0 & 36.2 \end{bmatrix}$

piezoelectric coefficients (C m^{-2}) = $\begin{bmatrix} 0 & 0 & 0 & 0 & -0.545 & 0 \\ 0 & 0 & 0 & -0.510 & 0 & 0 \\ -0.525 & -0.633 & 0.636 & 0 & 0 & 0 \end{bmatrix}$

Polar optical phonon frequency (THz) = 8.46

3 Convergence tests of interpolation meshes for electronic transport properties

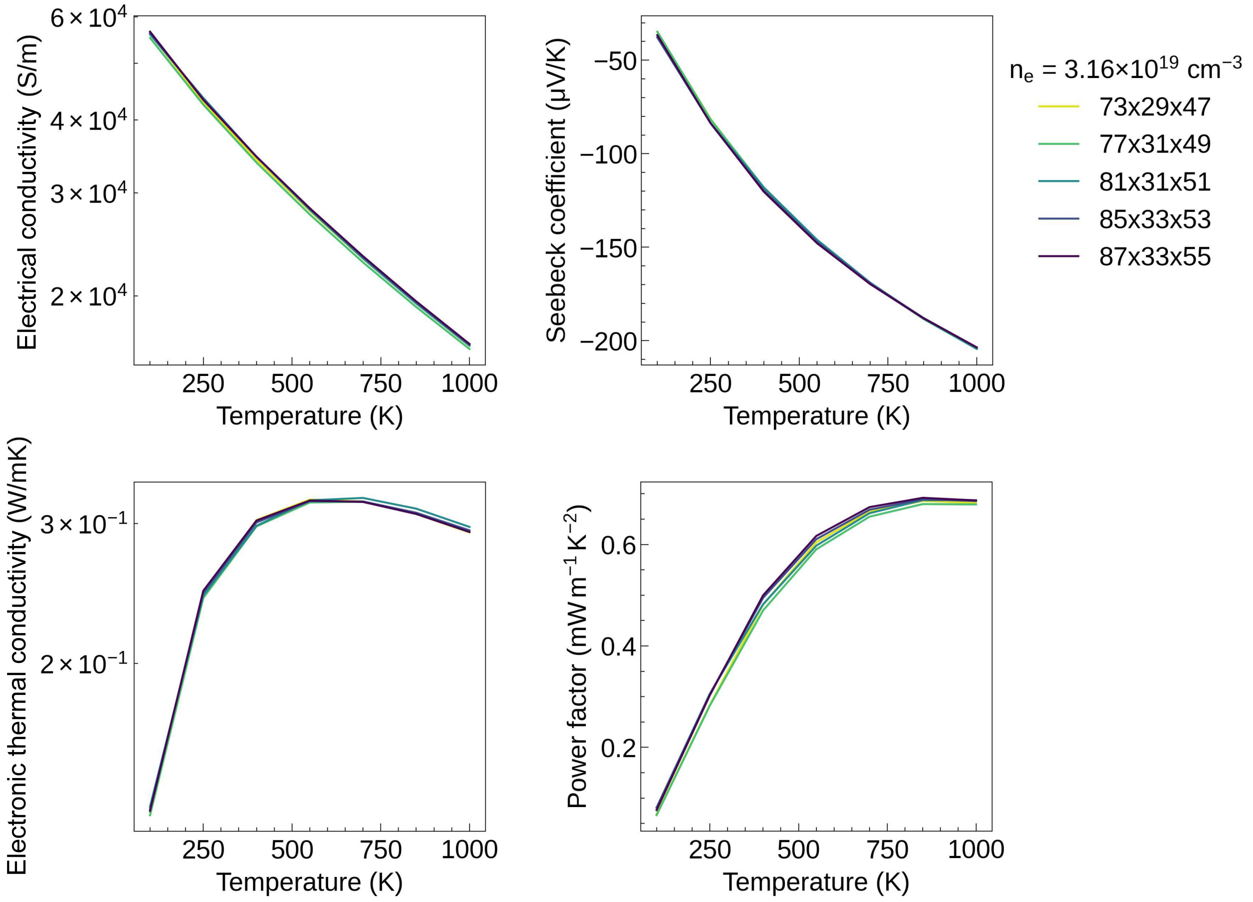

Figure S2. Temperature-dependent electronic transport properties of $SrZn_2S_2O$ calculated using different interpolated meshes, including electrical conductivity, Seebeck coefficient, electronic thermal conductivity, and power factor.

4 Convergence tests of phonon supercell meshes

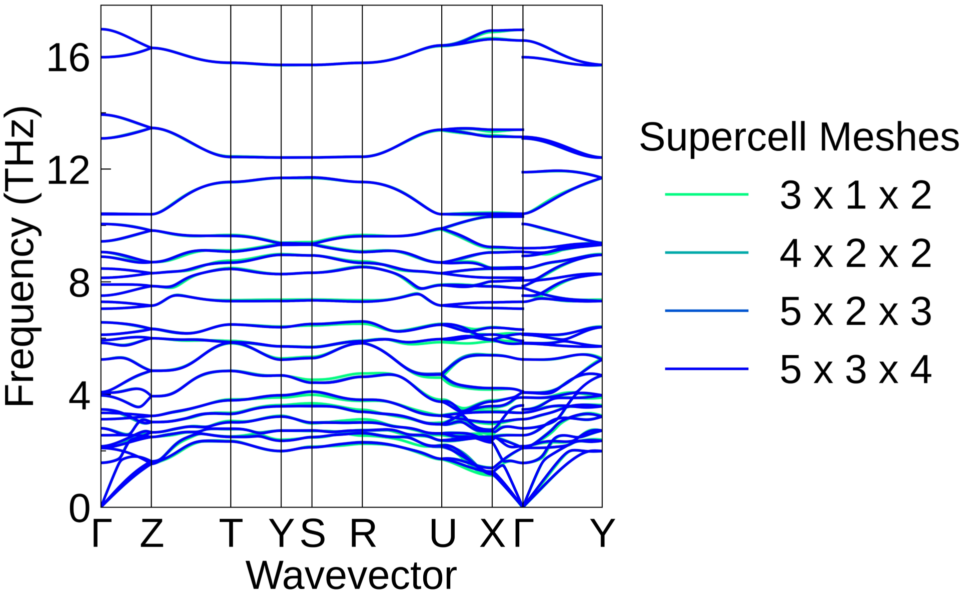

Figure S3. Phonon dispersions of $SrZn_2S_2O$ calculated using different supercell meshes, plotted using ThermoParser.[1] The high-symmetry path was constructed based on the Bradley-Cracknell formalism.[2]

5 Convergence tests of lattice thermal conductivity with respect to *q*-point sampling meshes

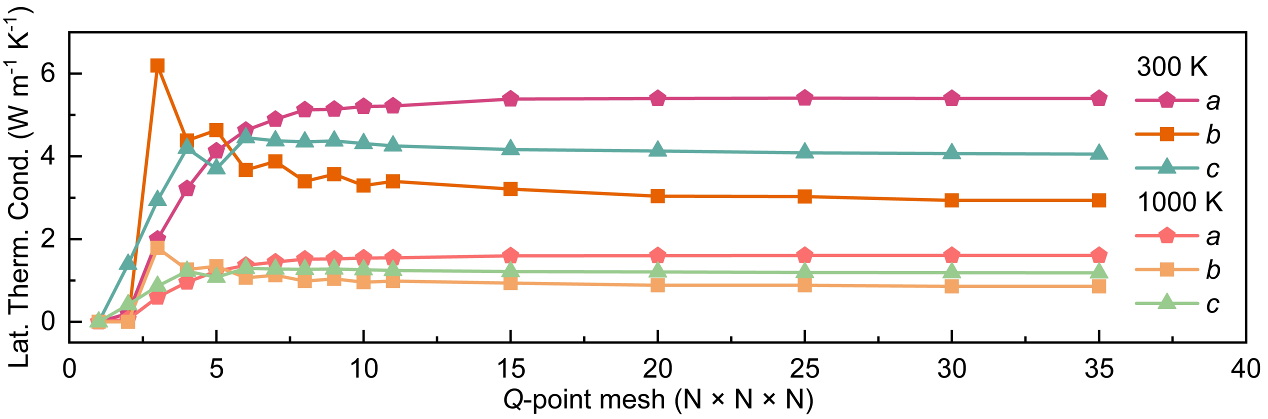

Figure S4. Lattice thermal conductivity of $SrZn_2S_2O$ calculated using different *q*-point sampling meshes.

6 Calculated electrical conductivity and Seebeck coefficient of $SrZn_2S_2O$ and BiCuSeO

Table S1. Calculated *n*-type electrical conductivity and Seebeck coefficient of $SrZn_2S_2O$ and BiCuSeO at 300, 600, and 900 K with a carrier concentration of 1×10^{19} cm^{-3}.

System	Electrical conductivity (S m^{-1})			Seebeck coefficient (μV K^{-1})		
	300 K	600 K	900 K	300 K	600 K	900 K
$SrZn_2S_2O$	1.33×10^4	7.43×10^3	4.86×10^3	153	236	285
BiCuSeO	3.42×10^3	1.91×10^3	1.24×10^3	347	381	412

Table S2. Calculated *n*-type electrical conductivity and Seebeck coefficient of $SrZn_2S_2O$ and BiCuSeO at 300, 600, and 900 K with a carrier concentration of 1×10^{20} cm^{-3}.

System	Electrical conductivity (S m^{-1})			Seebeck coefficient (μV K^{-1})		
	300 K	600 K	900 K	300 K	600 K	900 K
$SrZn_2S_2O$	1.12×10^5	7.95×10^4	5.93×10^4	47	84.5	112
BiCuSeO	3.88×10^4	2.17×10^4	1.31×10^4	212	237	256

Table S3. Calculated *p*-type electrical conductivity and Seebeck coefficient of $SrZn_2S_2O$ and BiCuSeO at 300, 600, and 900 K with a carrier concentration of 1×10^{19} cm^{-3}.

System	Electrical conductivity (S m^{-1})			Seebeck coefficient (μV K^{-1})		
	300 K	600 K	900 K	300 K	600 K	900 K
$SrZn_2S_2O$	2.41×10^3	7.15×10^2	3.32×10^2	285	400	483
BiCuSeO	9.31×10^2	3.66×10^2	1.64×10^2	450	526	548

Table S4. Calculated *p*-type electrical conductivity and Seebeck coefficient of $SrZn_2S_2O$ and BiCuSeO at 300, 600, and 900 K with a carrier concentration of 1×10^{20} cm^{-3}.

System	Electrical conductivity (S m^{-1})			Seebeck coefficient (μV K^{-1})		
	300 K	600 K	900 K	300 K	600 K	900 K
$SrZn_2S_2O$	2.36×10^4	1.01×10^4	5.13×10^3	113	212	289
BiCuSeO	1.00×10^4	3.68×10^3	1.59×10^3	291	332	376

All calculated data for the electrical conductivity and Seebeck coefficient of BiCuSeO were taken from Ref. 3.

7 Cumulative lattice thermal conductivity

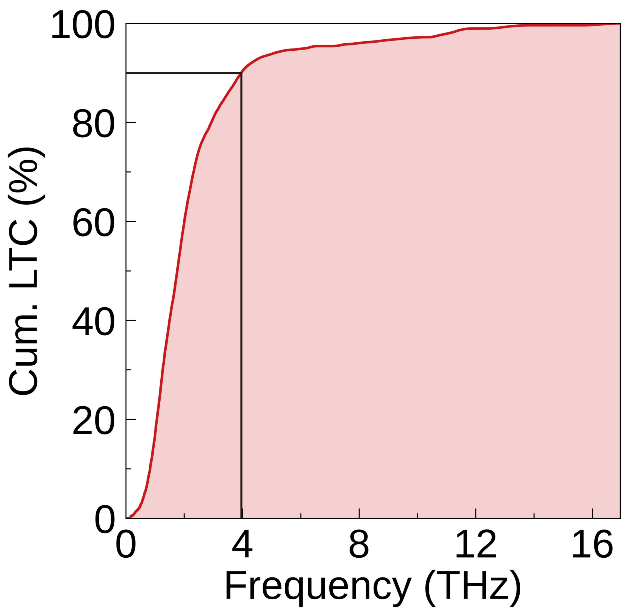

Figure S5. Cumulative lattice thermal conductivity of $SrZn_2S_2O$ as a function of phonon frequency at 300 K. The horizontal line marks 90% of the cumulative lattice thermal conductivity, and the vertical line indicates the corresponding frequency.

8 Anisotropic *ZT*

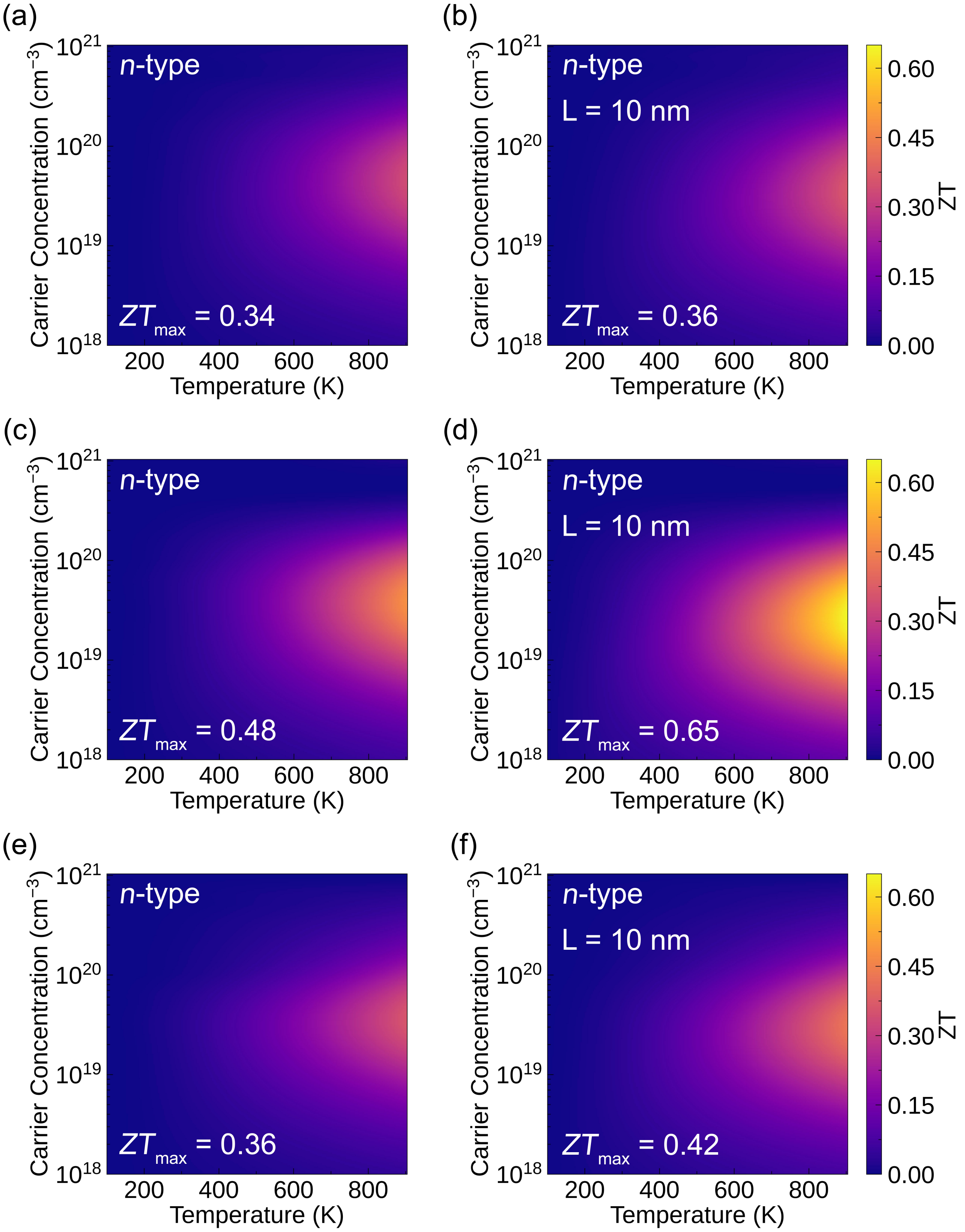

Figure S6. Calculated *n*-type *ZT* values of $SrZn_2S_2O$ as functions of temperature and carrier concentration. (a), (c), and (e) show the intrinsic *ZT* along the *a*, *b*, and *c* directions, respectively, while (b), (d), and (f) present the *ZT* of the nanostructured system along the same directions. The analysis was performed using ThermoParser.[1]

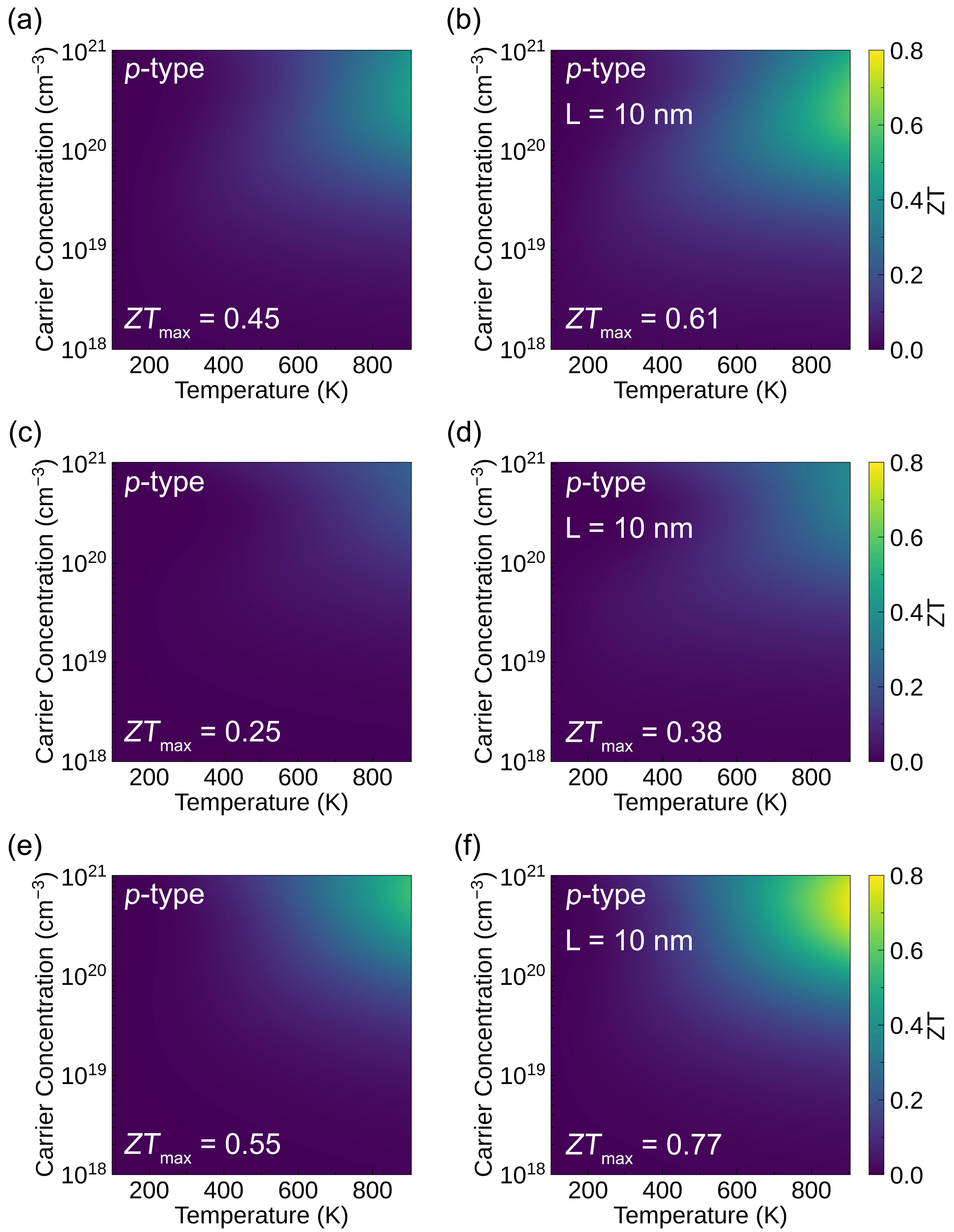

Figure S7. Calculated *p*-type *ZT* values of $SrZn_2S_2O$ as functions of temperature and carrier concentration. (a), (c), and (e) show the intrinsic *ZT* along the *a*, *b*, and *c* directions, respectively, while (b), (d), and (f) present the *ZT* of the nanostructured system along the same directions. The analysis was performed using ThermoParser.[1]

REFERENCES

(1) Spooner, K. B.; Einhorn, M.; Davies, D. W.; Scanlon, D. O. ThermoParser: Streamlined Analysis of Thermoelectric Properties. *J. Open Source Softw.* **2024**, *9*, 6340.

(2) Bradley, C. J.; Cracknell, A. P. *The Mathematical Theory Of Symmetry In Solids: Representation theory for point groups and space groups*; Oxford University Press: Oxford, **2009**.

(3) Wang, N.; Li, M. L.; Xiao, H. Y.; Gao, Z. B.; Liu, Z. J.; Zu, X. T.; Li, S. A.; Qiao, L. Band degeneracy enhanced thermoelectric performance in layered oxyselenides by first-principles calculations. *npj Comput. Mater.* **2021**, *7*, 18.
